# Programmable soliton dynamics in all-Josephson-junction logic cells and networks

**DOI:** 10.3762/bjnano.16.131

**Published:** 2025-10-28

**Authors:** Vsevolod I Ruzhickiy, Anastasia A Maksimovskaya, Sergey V Bakurskiy, Andrey E Schegolev, Maxim V Tereshonok, Mikhail Yu Kupriyanov, Nikolay V Klenov, Igor I Soloviev

**Affiliations:** 1 Lomonosov Moscow State University, Skobeltsyn Institute of Nuclear Physics, Moscow, 119991, Russia; 2 All-Russian Research Institute of Automatics n.a. N.L. Dukhov (VNIIA), 127030, Moscow, Russia; 3 Lomonosov Moscow State University, Faculty of Physics, Moscow, 119991, Russia; 4 Moscow Institute of Physics and Technology, 141700 Dolgoprudny, Russia; 5 Moscow Technical University of Communications and Informatics (MTUCI), 111024, Moscow, Russia

**Keywords:** Josephson-based diode, kinetic inductance, soliton dynamics, superconducting electronics, superconducting neural networks

## Abstract

We demonstrate the programmable control of kinetic soliton dynamics in all-Josephson-junction (all-JJ) networks through a novel tunable cell design. This cell enables on-demand switching of transmission lines and operates across defined parameter regimes supporting diverse dynamical modes. By introducing a structural asymmetry into a transmission line, we implement a Josephson diode that enforces unidirectional soliton propagation. The programmability of the kinetic inductance then provides a crucial mechanism to selectively enable or disable this diode functionality. By engineering artificial inhomogeneity into the circuit architecture, we enhance robustness in all-JJ logic circuits, 2D transmission line all-JJ lattices, and neuromorphic computing systems.

## Introduction

The rapid advancement of Josephson junction (JJ) logic circuits [1–5] and neuromorphic networks [6–9] holds transformative potential for ultra-low-power computing. However, achieving scalable integration remains a critical bottleneck, as conventional JJ-based architectures face fundamental density constraints imposed by magnetic flux manipulation requirements and complex mutual inductive crosstalks.

Circuits composed entirely of Josephson junctions (all-JJ circuits) [10–16] represent a promising platform for energy-efficient, high-speed, and scalable computing. In these systems, the propagation of information is associated with the movement of a current wave/topological soliton, which is clearly visible in the model by a 2π jump of the so-called Josephson phase, φ. In contrast to conventional rapid single flux quantum (RSFQ) logic, the phase drop for the considered single kinetic soliton (SKS) occurs not on the relatively large connecting geometric inductors, but on the Josephson junctions. SKS is a propagating wave of phase change with kinetic energy limited from below; the corresponding current pulse “dissipates” if its motion is interrupted, for example, by a structural inhomogeneity in a transmission line. Traditionally, this sensitivity to structural inhomogeneities has been viewed as a challenge for robust circuit design.

In this work, we propose to exploit the sensitivity mentioned above. We base our proposal on the concept of applying a small number of key cells, which should create precisely engineered tunable inhomogeneities. Such inhomogeneity may be designed as an element of tunable kinetic inductance [17]. This element has high inductance at small scales and can be controlled using currents [18–19], voltage [20], or magnetic fields [21–22]. At the same time, the use of hybrid superconductor–normal metal structures makes it possible to increase the effect of frequency tuning [23–24], while the addition of ferromagnetic layers permits the non-volatile control [25–26]. Another feature of tunable kinetic inductance element is the linear behavior for weak signals, which excludes formation of parasitic processes in the transmission line. This permits to apply tunable kinetic inductance in the resonators with shifting resonance frequency [19,21–22], as well as in sensitive all-JJ digital circuits.

This idea enables us to use the “flaws” of the structure as its important features, opening up a pathway to creating programmable and reconfigurable large circuits. An obvious and widely required application of this technology is in the development of superconductive programmable gate arrays (SPGAs) [27–30], an active area of current research. Another important application of this idea lies in the promising neuromorphic direction [31–33]. Earlier in [34], we have already proposed using kinetic inductances to control neuron dynamics in networks based on radial basis functions (RBF-networks). Moreover, this approach can be extended to hardware realizations of bio-inspired spiking neural networks [35–42] by solving the challenges of creating controllable synapses to realize the effect of spike-timing-dependent plasticity and unidirectional feedbacks for self-regulation. Furthermore, the physical resemblance between solitons and the action potentials (spikes of voltage) generated in biological nervous systems makes all-JJ structures tempting candidates for constructing neuromorphic hardware [43].

In this paper, we investigate the use of controlled kinetic inductance to create an engineered inhomogeneous medium for kinetic solitons. We demonstrate that by tuning this inhomogeneity, distinct dynamical modes can be induced, fundamentally altering the soliton’s behavior. Furthermore, we explore how structural asymmetry within this medium can be exploited to achieve a diode effect, enabling non-reciprocal soliton propagation. Building upon these foundational concepts, we then propose two specific architectural solutions: a programmable switch and a versatile routing matrix, which we term the “WayMatrix”. We suggest that these architectures provide a framework for the flexible configuration of advanced logic and neuromorphic circuits.

## Results

### Model description

To model the dynamics of kinetic solitons [43], we employ the resistively and capacitively shunted junction (RCSJ) model [1], where the total current *I* across a Josephson junction is the sum of the supercurrent, the quasi-particle current, and the displacement current:


[1] $$I=I_{\text{c}}\sin\left(\varphi \right)+\frac{V}{R_{\text{N}}}+C\frac{\text{d}V}{\text{d}t}.$$

Here, φ is the phase difference for the complex superconducting order parameter across the junction, *V* is the voltage, *I*_c_ is the critical current, *R*_N_ is the resistance in the normal state and *C* is the capacitance. For analysis, it is convenient to express this equation in a dimensionless form. We normalize the time to the inverse of a reference plasma frequency, 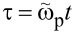, where 
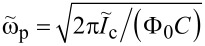
, and normalize the current to a reference critical current 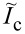. This yields:


[2]

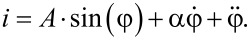



In this normalized equation, the dots above the phases indicate differentiation over time with respect to τ. The dimensionless damping coefficient is 
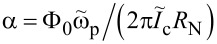
. The term 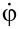 represents the voltage normalized by the characteristic voltage 
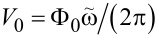
. The parameter 
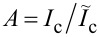
 is the normalized amplitude of the critical current for junctions with the critical current *I*_c_ that differs from the reference normalization value 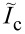.

To analyze the circuit dynamics, we adopt a nodal analysis approach. In this approach, the gauge-invariant phase difference across any element is expressed in terms of the nodal phases at its terminals, φ = φ*_k_* − φ*_j_*. The phase of the ground node is set to zero by convention. This formulation inherently satisfies Kirchhoff’s current law (KCL) at each node. For any node *k* connected to *H* elements, KCL dictates that the algebraic sum of currents is zero:


[3] $$\sum_{h=1}^{H}I_{k,j\left(h\right)}=0,$$

where the index *h* runs over all elements connected to node *k*, *j*(*h*) is the index of the node at the other end of element *h*, and *I_k_*_,_*_j_*_(_*_h_*_)_ is the normalized current flowing from the node *k* to the node *j*(*h*). Each current is described by the RCSJ model (Equation 2):


[4]

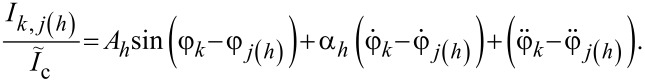



With this approach, the current across the inductance is defined by the expression


[5]

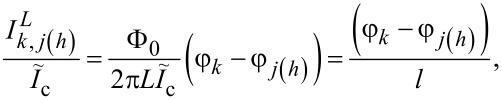



where *l* = *L*/*L*_J_ is inductance normalized to the Josephson inductance 
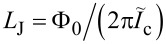
.

After substituting the expressions for the current into the formula for the current balance at the node, we get:


[6]

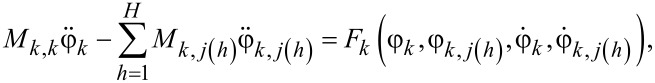



where *M_k,k_* is the sum of the coefficients before 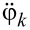, *M_k_*_,_*_j_*_(_*_h_*_)_ are the coefficients before 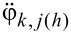, *F_k_* contains the sum of all summands except those that do not contain the second derivative. In *F_k_*, all summands with φ*_k_* are written with a minus sign, and all summands with φ*_k_*_,_*_j_*_(_*_h_*_)_ are written with a plus sign. Additional currents (e.g., the bias current or the time-dependent current from the generator) are also included as components. After writing down Equation 6 for each node, a system of second-order diffeomorphic equations are obtained, which can be represented in matrix form:


[7]

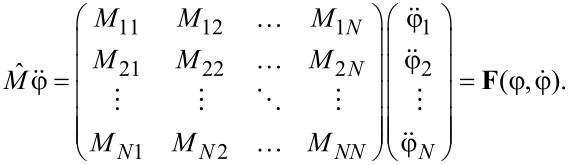



The resulting system of *N* ordinary differential equations is expressed in the matrix form shown in Equation 7. In this equation, 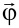 is the vector of nodal phases, *N* is the total number of non-ground nodes, and 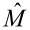 is the *N* × *N* mass matrix (also known as the capacitance matrix), which is defined by the capacitive coupling coefficients from Equation 6. A key property of 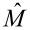 is its sparsity, which arises directly from the local connectivity of the circuit topology; each node is connected to a small subset of other nodes. To increase computational efficiency, we exploit this sparsity when solving the system. The equations are integrated numerically using an adaptive-step-size solver based on the explicit Runge–Kutta (4th and 5th order) formula, commonly known as the Dormand–Prince pair [44–45], which is well suited for this class of non-stiff problems.

**Figure 1:**
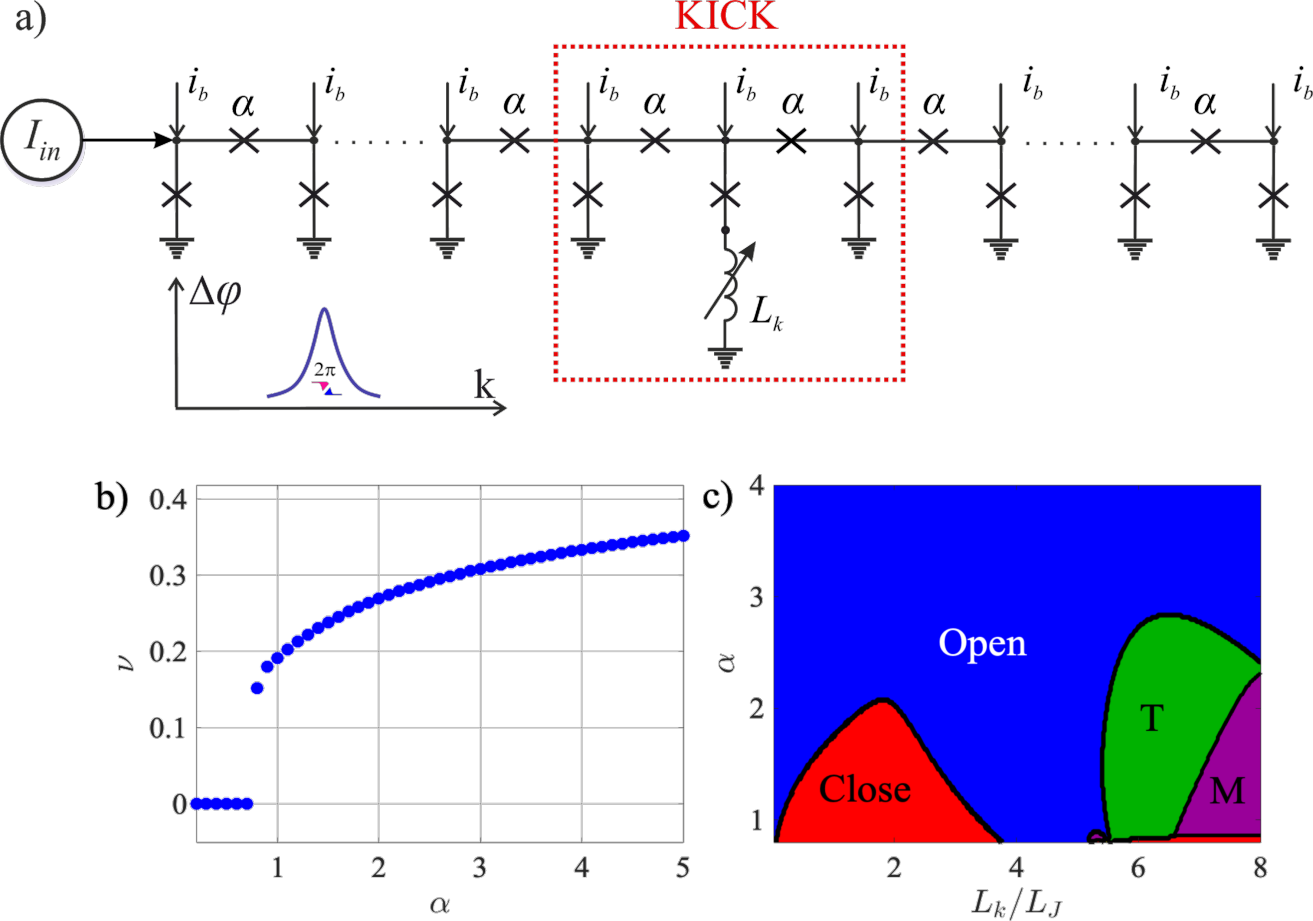
(a) An equivalent scheme for the “kinetic inductance controllable key” (KICK) as a part of an all-Josephson transmission line. A soliton whose dynamics is controlled by the developed key is represented schematically. (b) Dependence of the single kinetic soliton (SKS) propagation velocity, measured in Josephson junctions per normalized time unit, on the damping parameter α. (c) Map of different modes depending on the damping parameter and kinetic inductance measurement. Close mode (red zone): The KICK does not allow the SKS to pass through. Open mode (blue zone): The SKS passes through the KICK. T-mode (green zone): The KICK has two stable states, and every second SKS passes through it. M-mode (purple zone): The KICK has many stable states.

The fundamental building block of our design is the “kinetic inductance controllable key” (KICK), which is constructed from the two modified unit cells of an all-Josephson Junction Transmission Line (all-JJTL). As depicted in Figure 1a, each cell is modified by incorporating a controlled kinetic inductance in series with one of its Josephson junctions connected to the ground plane. There are some operational regimes inherent to such a KICK governed by the value of this inductance and by the damping parameter of junctions within the transmission line. The damping parameter is a critical factor as it dictates the kinetic soliton’s propagation rate.

As a preliminary step, we characterized the dependence of the kinetic soliton propagation velocity on the damping parameter of the connecting junctions, α (see Figure 1b). We define the velocity as the number of grounded junctions traversed per unit of normalized time, τ. Our simulations revealed a critical damping threshold at α_crit_ ≈ 0.8; below this value, stable soliton propagation is not supported. Also, under this condition, the energy dissipation rate is too high relative to the energy transfer between adjacent junctions, causing the soliton to decay. When α *>* α_crit_, the soliton velocity is a monotonically increasing function of the damping. This dependence falls into an approximately linear regime for α *>* 3. The physical mechanism for this velocity increase can be understood from the RCSJ model; a higher value of α enhances the resistive quasiparticle current (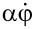) that flows as a junction switches. This larger current provides a stronger driving force to the next junction in the line, causing it to reach its critical threshold and switch more rapidly, thus increasing the overall propagation velocity of the soliton.

The functionality of the KICK is determined by the interplay between the damping α and the normalized kinetic inductance *L*/*L*_J_. Figure 1c summarizes the behavior of the device in a parameter map, which reveals four distinct operational regimes: (1) Open mode: The KICK is effectively transparent, allowing an incident kinetic soliton to propagate through it with minimal perturbation. (2) Close mode: The KICK acts as a terminator, blocking and destroying the incoming soliton. (3) T-mode: The KICK functions as a T-flip-flop. It possesses two stable states, and each arriving soliton toggles the cell from its current state to the other. Every second soliton passes to the exit. (4) M-mode (Multistate mode): This regime is characterized by the formation of more than two stable states and other complex dynamics, which fall outside the scope of this study.

An essential feature of the KICK is the ability to switch between different modes at a fixed value of α; thus, by fixing α (e.g., α = 2) and varying the kinetic inductance, we can switch between all modes (Open mode → Close mode → Open mode → T-mode → M-mode) represented on the parameter map (see Figure 1).

**Figure 2:**
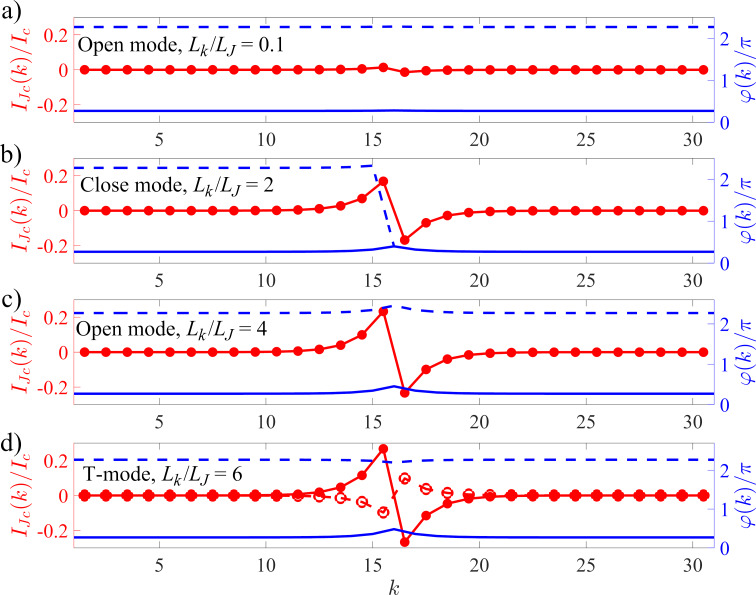
Spatial distributions of the Josephson phase (blue curves) and current (red curves) in (a, c) Open mode, *L_k_*/*L*_J_ = 0.1 (a) and *L_k_*/*L*_J_ = 4 (c); (b) Close mode, *L_k_*/*L*_J_ = 2; and (d) T-mode, *L_k_*/*L*_J_ = 6. Solid lines show initial profiles, and dashed lines represent distributions after soliton passage. The Josephson phase is plotted against the integer node index *k*, whereas the current is plotted at the midpoint index *k* + 0.5 to represent the junction between nodes *k* and *k* + 1, see Figure 1a.

To illustrate the operational modes of the KICK, we simulated the propagation of a kinetic soliton through the all-JJTL. The simulated line comprises 31 grounded junctions with a uniform damping parameter of connecting junctions α = 1. The KICK is implemented by inserting a controlled kinetic inductance in series with the ground junction at the line’s center (node *k* = 16). Figure 2 presents the results for different values of this inductance, corresponding to distinct operational modes. Each panel displays two key physical quantities on dual *y*-axes: (1) the spatial profile of the nodal Josephson phases (φ*_k_*) as a function of the node index *k* and (2) the normalized currents flowing through the series junctions connecting the nodes. The current between nodes *k* and *k* + 1 (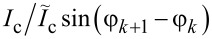) is plotted at the midpoint index *k* + 0.5 for visual clarity.This visualization allows for a direct comparison of the system’s state before and after soliton interaction. The solid lines depict the initial state (before the soliton reaches the KICK), and the dashed lines show the final state (after the soliton has passed and the system has settled).

For a low inductance of *L*/*L*_J_ = 0.1 (see Figure 2a), corresponding to the Open mode, the KICK causes only slight disturbance in the transmission line.The incident soliton propagates through it unimpeded, and the entire line returns to its initial physical state. However, increasing the inductance to *L*/*L*_J_ = 2 (see Figure 2b) switches the system to the Close mode. In this mode, the KICK serve as a significant barrier; when the soliton arrives, the large inductance impedes the necessary current dynamics, halting the propagation and causing the soliton to be annihilated. Consequently, the 2π phase slip, which signifies the soliton’s passage, only traverses the first half of the line (nodes 1 to 15), while the segment beyond the KICK remains entirely unperturbed. Remarkably, a further increase of inductance to *L*/*L*_J_ = 4 (see Figure 2c) leads to the re-emergence of the Open mode. This non-trivial effect is governed by transient energy storage in the inductor *L*. Although the soliton is momentarily halted at the KICK, the subsequent release of stored magnetic energy provides the necessary “kick” to complete the phase slip at node 16. This re-initiates the propagation, allowing the soliton to effectively re-form and travel down the rest of the line. Similarly to the low-inductance case, the soliton successfully traverses the entire line, and the system returns to its initial physical state.

The behavior of the KICK in the T-Mode, which enables its use as a T-flip-flop, is detailed in Figure 2d. This mode is defined by the existence of two distinct stable states, physically corresponding to a bistable potential landscape created by the KICK architecture. These two states are distinguished by the presence of persistent, static currents of opposite polarity flowing from the central node (*k* = 16). This physical difference leads to an fundamentally state-dependent and asymmetric toggling action. When the KICK is in the first stable state, an incoming soliton successfully flips it to the second state and is transmitted, continuing its propagation down the line. Conversely, when starting from the second state, an arriving soliton again flips the KICK back to the first state, but it is annihilated in the process and does not propagate further. This state-dependent transmission and annihilation is the core mechanism that allows the KICK to function as a memory element or a dynamic routing switch.

Beyond primary operational modes, the system exhibits other notable behavior types in specific regions of its parameter space. The M-Mode, for instance, is characterized by complex responses, depend on previous events. This can include such behavior when an initial soliton is annihilated, effectively “priming” the cell to transmit all subsequent solitons, a feature potentially useful for tasks like sequential filtering. Furthermore, in the transition regions between the primary modes, we observe phenomena such as soliton reflection back towards the source.

Finally, the asymptotic behavior in the high-damping (α) limit is particularly significant. As α increases, so does the soliton’s velocity and kinetic energy. Consequently, for sufficiently high α, the soliton’s energy is large enough to overcome any potential barrier presented by the KICK, ensuring transmission regardless of the inductance value. This results in a universal Open mode at high rates. Crucially, this high-energy passage is not inert; if the KICK is in a bistable regime (such as the T-Mode), the “passing” soliton can still deliver enough of an impulse to toggle the cell’s state.

### The soliton diode

What is even more interesting is that the KICK architecture can be engineered to function as a soliton diode, a device the function of which is similar to that of a semiconductor diode, allowing the soliton to pass in only one direction. This is achieved by introducing a structural asymmetry into the cell’s design. It is important to note that such non-reciprocal behavior can be achieved even without the kinetic inductance (*L* = 0). However, the inclusion of one (i.e., a tunable inductance) is a key innovation, as it allows to dynamically switch this directional property on and off.

We demonstrate this principle through simulation of a KICK with *L*/*L*_J_ = 2. In our model, the transmission line’s series junctions have a nominal critical current of 
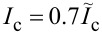
. The asymmetry is created by increasing the critical current of the specific junction connecting nodes 15 and 16 to 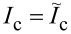 (i.e., to 1 in normalized units). The effect of this asymmetric potential barrier is that a soliton initiated in the forward direction (from node 1) successfully overcomes it and is transmitted along the entire line. In contrast, a soliton propagating in the reverse direction (from node 31) is unable to pass the barrier and is annihilated at node 17.

**Figure 3:**
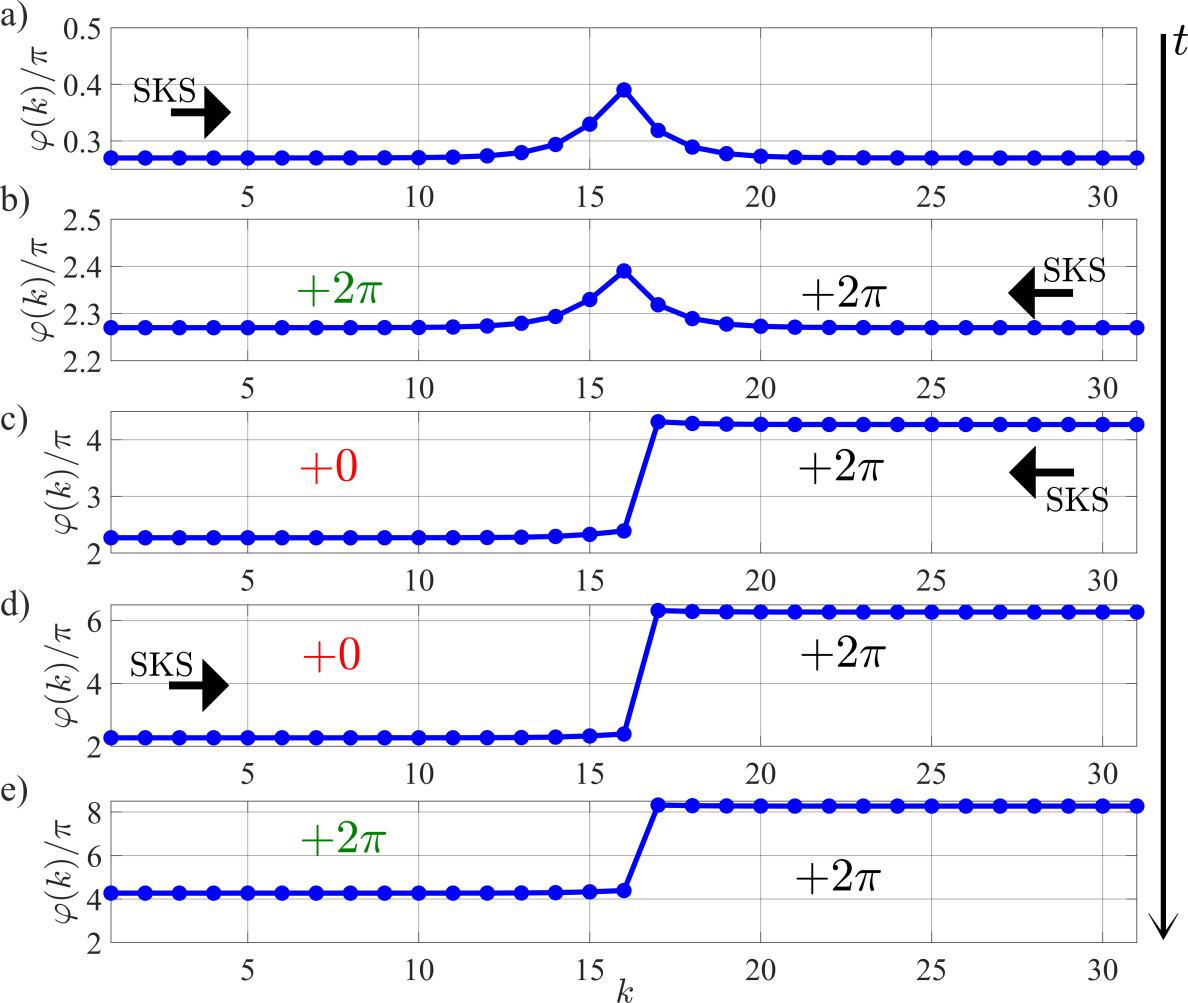
Temporal evolution of Josephson phase asymmetry in a soliton diode: (a) initial state; (b) after left-propagating soliton passage; (c, d) sequential right-propagating soliton interactions; (e) final left-propagating soliton recurrence.

Figure 3 demonstrates the non-reciprocal behavior of the soliton diode by showing a sequence of five snapshots of the nodal Josephson phase distribution at successive moments in time, arranged from top to bottom. The process begins with the line in its initial state (top panel), after which a soliton is initiated from the left side (node 1). As shown in the second panel, this forward-propagating soliton successfully passes through the diode, resulting in a 2π phase advance across all nodes. Immediately after, a new soliton is initiated from the right side (node 31) to test the reverse direction. The third panel reveals that this soliton is blocked; its propagation is halted at the diode, and the corresponding 2π phase slip is confined to nodes 17 through 31. The fourth panel confirms the robustness of this blocking action, as a second, subsequent reverse-propagating soliton is also annihilated in the same manner. To complete the demonstration, another forward-propagating soliton is sent from the left. The fifth panel confirms that the diode once again allows it to pass, resulting in another full 2π phase advance across the entire line. It is crucial to note that although the absolute phase values accumulate in multiples of 2π throughout this sequence, the physical state of the structure remains unchanged after each full transmission, a direct consequence of the 2π periodicity of the Josephson energy.

A significant feature of this structure is the ability to disable the diode effect. By increasing the inductance to *L*/*L*_J_ = 3, the device becomes bi-directionally transparent, effectively turning the diode function “off”. This demonstrates how the introduced structural asymmetry alters the operational landscape of the device: An inductance value that would normally correspond to the Close mode in a symmetric KICK now matches to a bi-directional Open mode for the asymmetric diode structure. Furthermore, it is worth noting that the asymmetry required for diode-like behavior can be achieved through alternative means, such as by creating a local mismatch in the damping parameter, for instance, by increasing α from 1 to 3 for one of the series junctions instead of the critical current.

The ability to enforce a specific direction of soliton flow makes the soliton diode an essential component for complex circuit design. This is particularly critical in architectures involving feedback loops, where it is necessary to unambiguously define the direction of signal propagation. This concept can be extended by cascading two such tunable diodes with opposing forward directions. This configuration creates a programmable transmission line where the permitted direction of soliton travel can be pre-configured by setting the inductance values of each diode.

## Discussion

### Implementation of reconfigurable networks

On the basis of the operational principles of the kinetic inductance controllable key and the soliton diode, we now demonstrate how these fundamental building blocks can be integrated to create reconfigurable soliton-based logic circuits. We begin by proposing a specific proof-of-concept design for a signal routing network and then introduce a generalized, scalable architecture suitable for complex computational tasks.

**Figure 4:**
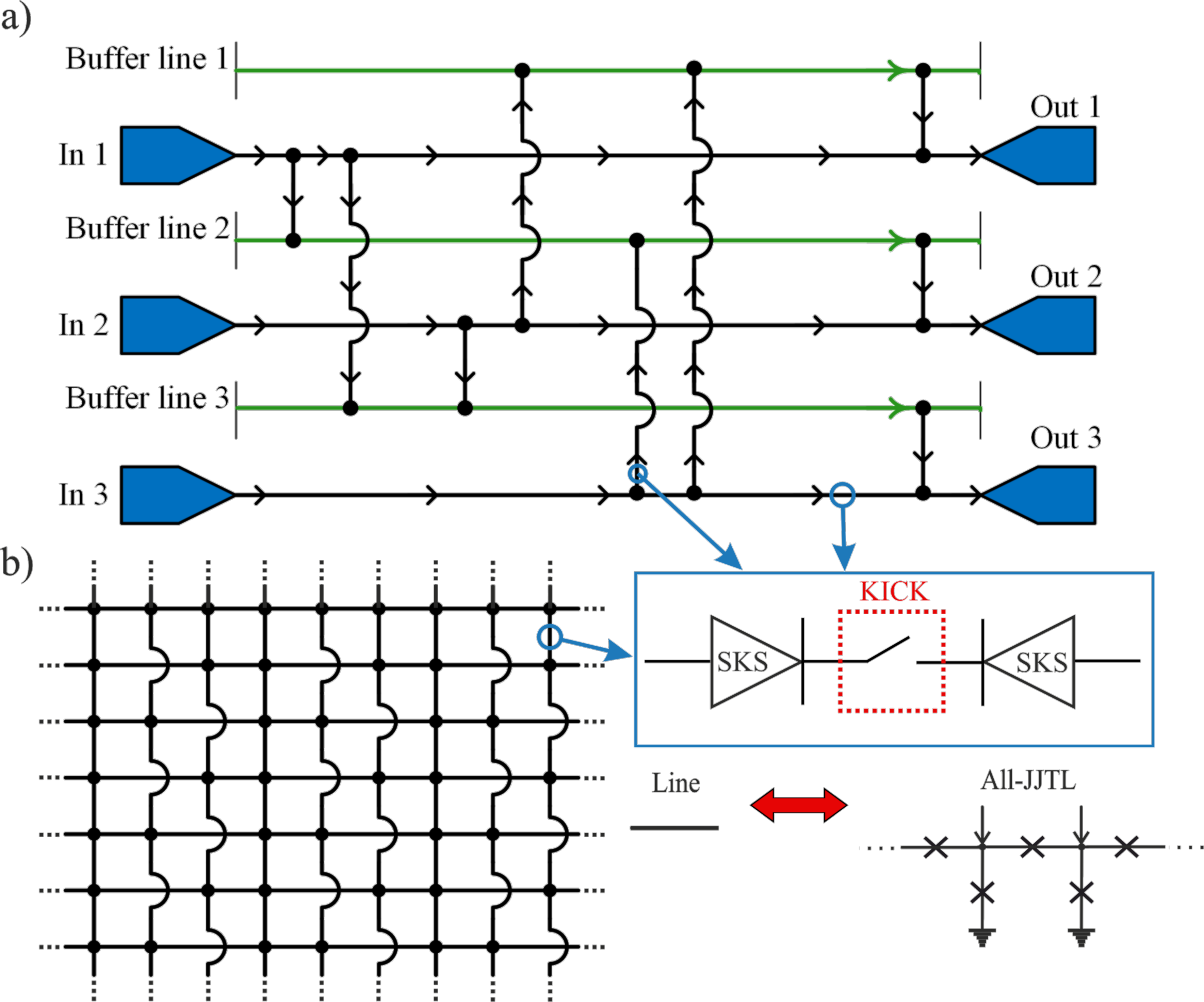
(a) Schematic of an all-Josephson-junction transmission line (all-JJTL) network with three inputs (In 1, In 2, In 3), three outputs (Out 1, Out 2, Out 3), and three auxiliary buffer lines. Black arrows on the lines indicate soliton propagation paths. Input–output connections are configured by setting operation modes of kinetic inductance controllable keys (KICKs), where each cell either transmits or blocks solitons based on its programmed state. (b) The schematic shows a transmission line matrix where path selection is governed by KICKs and signal directionality is ensured by soliton diodes. Specific vertical lines enable row-skipping connections to prevent soliton collisions during signal propagation.

As a direct application of the KICK’s switching capabilities, we first propose the three-input, three-output routing network illustrated in Figure 4a. The proposed architecture is based on a grid where each path depicted is itself a complete all-JJTL. The routing mechanism would depend on the incorporation of KICKs into specific segments of these all-JJTLs. By programming each KICK to be in either its Open mode (transmitting) or Close mode (blocking), one could control the flow of solitons through the network and define a unique path from any input to any output. To prevent collisions between solitons traveling along different routes, the design incorporates auxiliary buffer lines. These lines make it possible to define a set of non-intersecting paths for all required connections, thus ensuring collision-free operation. This design serves to validate the fundamental principle of using KICKs as programmable switches.

With this idea, we propose a more general and powerful architecture, which we term the “WayMatrix”, shown schematically in Figure 4b. This versatile *N* × *M* routing matrix is conceived as a core component of larger soliton-based processors. Its enhanced functionality would be predicated on the synergistic action of its core components. First, KICKs integrated into the line segments would act as programmable switches controlling the signal flow. Second, the directionality of soliton propagation would be rigorously enforced by integrated soliton diodes. Thus, the diodes and switches placed in the all-JJTL lines determine the direction of soliton propagation in the line. Finally, to solve the problem of collisions in a dense matrix, we propose dedicated vertical lines that enable row-skipping connections. For the same purposes, horizontal lines can also be used for column-skipping connections.

At first glance, it may seem that the proposed architecture is a complicated version of a memristive crossbar, but this is not the case. The main distinction is in the organization of interconnections between lines: In a memristive crossbar, as the name suggests, these connections are formed by the intersection of signal lines and the corresponding memristive layer. In the proposed WayMatrix, however, the lines are combined into a single node at the intersection point, the current direction of which can be controlled by switches and diodes. The power of the WayMatrix architecture lies in its potential use as a universal framework for creating programmable and reconfigurable connections between different circuit blocks. WayMatrix makes it easy to set up feedback loops between these blocks, change their connection order, and perform logical operations. We envision it serving as a reconfigurable “backbone” to link various specialized functional units within a larger integrated circuit. For example, the WayMatrix could be configured to connect arrays of memory cells to arithmetic logic units or to route data between different processing cores. Another key application is the creation of programmable clock distribution networks. In such a role, the WayMatrix could manage signal timing across a chip by introducing precise, configurable delays into the clock paths, which is crucial for asynchronous circuit design. This would allow a single hardware platform to be flexibly repurposed for different algorithms by simply re-programming the routing paths, a paradigm central to the development of SPGAs.

The true potential of this architecture, however, is most evident in its application as an axon-synaptic connection matrix for neuromorphic computing. The ability to program connections, enforce directionality, and reconfigure paths makes the WayMatrix an ideal candidate for emulating the complex and plastic connectivity of a biological neural network. In such a system, each soliton acts as a “spike”, and the WayMatrix serves as the synaptic network that routes these spikes between artificial neurons. This lays the groundwork for building powerful, event-driven, and energy-efficient spiking neural networks based on the principles we have outlined. In addition to using the WayMatrix, we can reconfigure the neural network itself, program connections between different neurons, implement synaptic pruning, and even “kill” parts of the artificial brain.

Human or animal brains contain a huge number of synapses, many times greater than the number of neurons (e.g., the Norwegian rat brain contains about 200 million neurons, each of which roughly has an average of about 1000 synapses [46]). The ability of a living being to solve certain tasks depends precisely on the number of interneuronal connections. In their attempts to implement such complex systems in hardware, engineers and scientists inevitably face the problem of interconnects and the implementation of a huge number of synaptic connections. The superconducting axon-synaptic matrix based on the WayMatrix concept seems to be a promising solution to the problem [47–50].

As mentioned above repeatedly, the field applications of kinetic inductance and, in particular, KICK, also extend to bio-inspired neuromorphic spiking networks. One important feature of living nervous tissues is the ability to modulate the synaptic delay of signal propagation from one neuron to another. This feature is equally important to implement in hardware artificial realizations of neuromorphic networks. The signal propagation delay is also affected by a length and a conductivity of an axon, which is quite simply imitated by means of a standard Josephson transmission line, as well as by means of all-JJTLs, discussed at the beginning of this article. A simple solution to modulate the propagation delay is to change the length (number of JTL cells) of such an artificial axon, but there is another way. The inductance connected in parallel with the Josephson junctions determines the amount of magnetic energy stored within each JTL cell. Consequently, a larger inductance value results in a longer propagation delay.

## Conclusion

This study demonstrates the programmable control of kinetic soliton dynamics in all-Josephson-junction networks through a novel tunable element, the “kinetic inductance controllable key” (KICK). By engineering inhomogeneity via controlled kinetic inductance, we induce distinct dynamical modes (Open mode, Close mode, and T-mode) that fundamentally alter soliton propagation. Furthermore, the features of the proposed cell enable a soliton diode effect, achieving non-reciprocal signal transmission. Building on these principles, we propose two scalable architectures, namely, a programmable switch for reconfigurable routing and the WayMatrix, a versatile *N* × *M* routing matrix. These solutions establish a framework for robust, high-speed superconducting logic that addresses critical bottlenecks in this type of computing.

We realize that the time required to “reprogram” kinetic inductance significantly exceeds the picosecond timescales of Josephson junction dynamics. However, this re-configuration time should be considered in the context of hardware development cycles. From this point of view, the re-configuration time is orders of magnitude lower than the time required to design, fabricate, and test a new application-specific integrated circuit (ASIC), offering a compelling advantage in flexibility and prototyping rate.

The superconducting diodes proposed in this work can be used as a part of synaptic connections in neuromorphic networks to prevent the backward influence of a postsynaptic neuron on a presynaptic neuron through the same connection link. It should also be noted that the signal propagation time between neurons can be controlled by modulating the bias currents, the value of which directly affects the potential barrier in Josephson line (standard JTL or All-JJTL). Thus, the choice of a particular method of signal propagation delay influence depends on the realization of interneuron interactions and the need to adjust a particular interneuron connection. Moreover, these approaches can be combined into one by using a chain of superconductor diodes. Using cells with kinetic inductances, we can change the local propagation speed of spikes in interneuronal signal transmission circuits by smoothly adjusting the delay time. The integration of the WayMatrix will make it possible to change the length of the axonal line as a whole, and thus introduce a delay. Besides, it is really interesting to examine how the dynamics of voltage spike formation in a bio-inspired neuron, proposed in [42], will change if we substitute geometric inductances for kinetic ones. Further development of the idea presented in this article will also address this aspect.

The proposed technique allows for a more compact design and new (diode) functionality of various superconducting computing modules and makes possible further increase of integration density compared to well-known RSFQ technology.

## Data Availability

Data generated and analyzed during this study is available from the corresponding author upon reasonable request.
